# ESBL-producing and virulent enteric bacteria from goats in Northeastern Brazil

**DOI:** 10.1007/s11259-026-11102-w

**Published:** 2026-02-14

**Authors:** Guilherme Valeriano Silva, Denny Parente de Sá Barreto Maia Leite, Lucilene Martins Trindade Gonçalves, Pollyanne Raysa Fernandes de Oliveira, André de Souza Santos, Maria Aparecida Juliano, José Wilton Pinheiro Junior, Rinaldo Aparecido Mota

**Affiliations:** 1 Infectious and Contagious Diseases Laboratory, Department of Veterinary Medicine, Federal Rural University of Pernambuco, Recife, Pernambuco Brazil; 2 Departament of Biophysical, Paulista School of Medicine, Institute of Environmental, Chemical and Pharmaceutical Sciences, Federal University of São Paulo, São Paulo, SP Brazil; 3 Animal Virology Laboratory, Department of Veterinary Medicine, Federal Rural University of Pernambuco, Recife, Pernambuco Brazil

**Keywords:** Bacterial resistance, *Enterobacteriaceae*, ESBL, Production animals

## Abstract

The aim of this study was to identify *Enterobacteriaceae* in healthy milk, mastitic milk, and rectal swab samples from dairy goats and kids in herds located in the state of Pernambuco, Brazil, and to evaluate the phenotypic profile of ESBL-producing isolates, as well as to detect the genotypic expression of ESBL resistance genes and virulence factors. Rectal swab and milk samples were collected from 56 goats and 14 kids. Gram-negative bacillary colonies were identified using Matrix-Assisted Laser Desorption/Ionization Time-of-Flight Mass Spectrometry (MALDI-TOF MS). The double-disk synergy test (DDST) for the detection of extended-spectrum β-lactamases (ESBL) was performed using the antibiotic discs ceftazidime, ceftriaxone, cefotaxime, aztreonam, and amoxicillin/clavulanic acid. Genotypic profiling for ESBL and virulence factor genes targeted *blaSHV*, *blaTEM*, *blaCTX-M*, *stx1*, *stx2*, *eaeA*, and *hlyA*. *Escherichia coli*, *Enterobacter* spp., *Klebsiella* spp., and *Cronobacter* sp. were identified. The phenotypic test revealed that 27% (25/92) of the isolates were ESBL-positive. In the genotypic analysis, one isolate carried the *blaSHV* gene and one carried the *blaTEM* gene, while *blaCTX-M* was not detected. At least one virulence-associated gene (*hlyA*,* stx1*, and *stx2*) was detected in 32% (30/92) of the isolates. The detection of antimicrobial resistance and virulence factors in enteric bacteria from healthy goat herds highlights potential risks to human health and animal welfare, as well as possible economic losses in rural farming systems.

## Introduction

Goat farming represents a significant livestock activity in Brazil, generating income for thousands of farmers through the production of meat, milk, and leather. It serves as an essential source of nutrition and plays a key role in family-based agricultural development, thereby reinforcing its socioeconomic relevance. In the Northeast region of Brazil, goat farming constitutes a viable and sustainable option, as these animals are hardy, well-adapted to the semi-arid climate, and can be raised on smallholdings and in non-productive areas (De Souza Santos et al. 2023).

Enterobacteria are widely distributed in nature, occurring in soil, water, and vegetation, and also form part of the normal gastrointestinal microbiota of animals and humans (Koneman et al. 2018). Strains of *Escherichia coli* are commonly recovered from animal feces, while other genera, including *Klebsiella* spp. and *Enterobacter* spp., are often detected in soil, grains, and water. Although many enterobacteria are considered commensal, certain strains are recognized as important opportunistic pathogens, capable of causing mastitis in ruminants (Ninkovic et al. 2023), severe diarrhea (Whon et al. 2021) and represent an emerging threat to food safety (Gómez-Sanz et al. 2023).

Microorganisms within this family exhibit high genetic plasticity, which facilitates the acquisition and horizontal transfer of antimicrobial resistance genes and virulence factors, primarily via plasmids (Partridge 2015). Among the most clinically relevant resistance mechanisms are Extended-Spectrum Beta-Lactamases (ESBL) and carbapenemases, which confer resistance to β-lactam antibiotics commonly used in both human and veterinary medicine (Mahamat et al. 2021). In terms of virulence, the production of toxins and specific proteins, such as Shiga toxins, hemolysins, and intimin, constitutes a major threat to host health, contributing significantly to disease pathogenesis (Braz et al. 2020).

The aim of this study was to identify *Enterobacteriaceae* isolates in healthy milk, mastitic milk, and rectal swab samples from dairy goats and their offspring in herds located in the state of Pernambuco, Brazil. Furthermore, the study aimed to evaluate the phenotypic profile of ESBL-producing isolates and to detect the genotypic expression of ESBL resistance genes and virulence factors.

## Materials and methods

### Biological sample collection

Biological samples were collected from June to December 2024 on farms located in Northeast Region of Brazil, in the municipalities of Pernambuco state, Venturosa, Alagoinha, Cachoeirinha, Sanharó, and Gravatá. Samples included milk and rectal swabs collected from 56 lactating goats, totaling 112 milk samples, 56 rectal swab samples from adult goats, and 14 rectal swab samples from kids.

Milk samples were collected during routine milking with falcon-type tubes, following the protocol established by the National Mastitis Council (NMC 2001). Samples were properly labelled and, after collection, stored in insulated containers with recyclable ice packs and transported to the laboratory for processing. Fecal samples were obtained using sterile plastic-handled swabs in transport medium tubes.

### Isolation and identification of *Enterobacteriaceae*

Milk samples were cultured on MacConkey agar and blood agar base supplemented with 5% sheep blood and incubated aerobically at 37 °C for 24 to 48 h in a microbiological incubator. Rectal swab samples were processed for the identification of *Salmonella* spp. and other *Enterobacteriaceae* using an adapted protocol from the International Organization for Standardization (ISO 6579-1:2017/Amd 1:2020). All colonies with Gram-negative bacillary morphology were subsequently identified using Matrix-Assisted Laser Desorption/Ionization Time-of-Flight Mass Spectrometry (MALDI-TOF MS).

### Antibiotic susceptibility testing

To confirm ESBL production, the double-disc synergy test (DDST) was performed as described by Giske et al. (2013), using the antibiotic discs amoxicillin–clavulanic acid (30 µg), cefotaxime (30 µg), ceftazidime (30 µg), and cefepime (30 µg), placed between 20 and 30 mm apart to avoid false-negative results, as described by De Oliveira et al. (2020).

### Bacterial DNA extraction and detection of ESBL-encoding and virulence genes

Genomic DNA was extracted from bacterial colonies using the thermal lysis method described by Fan et al. (1995). DNA concentration and purity were subsequently assessed using a spectrophotometer (Thermo Fisher Scientific, Massachusetts, USA) by measuring absorbance at 260 nm.

Target genes included *blaSHV*, *blaTEM* (De Gheldre et al. 2003), and *blaCTX-M* (Pitout et al. 2004). To detect virulence factor genes, isolates were also subjected to multiplex PCR targeting *stx1*, *stx2*, *hlyA*, and *eaeA* (Paton and Paton 1998).

## Results

No cases of clinical mastitis were detected during physical examination or the strip cup test. Subclinical mastitis was identified in 44.64% of the goats (25/56). No *Enterobacteriaceae* were isolated from any of the 112 milk samples analyzed.

A total of 92 bacterial isolates were obtained from 70 rectal swabs, with 77.17*%* (71/92) originating from adult animals and 22.83*%* (21/92) from kids. A total of 76 isolates were identified as *Escherichia coli*, seven as belonging to the *Enterobacter* genus, six to the *Klebsiella* genus, and three as *Cronobacter* sp. (Table 1).

**Table 1:** Identification of *Enterobacteriaceae* species isolated from fecal samples of goats and kids in the state of Pernambuco, Brazil

Agent	Isolates	Goat Isolates	Kid Isolates
*Escherichia coli*	76 (82.60%)	57 (75.0%)	19 (25.0%)
*Klebsiella pneumoniae*	4 (4.34%)	4 (100%)	-
*Klebsiella variicola*	2 (2.17%)	2 (100%)	-
*Enterobacter cloacae*	3 (3.27%)	2 (66.67%)	1 (33.33%)
*Enterobacter asburiae*	3 (3.27%)	3 (100%)	-
*Enterobacter bugendensis*	1 (1.08%)	-	1 (100%)
*Cronobacter* sp.	3 (3.27%)	3 (100%)	-
Total	92 (100%)	71 (77.18%)	21 (22.82%)

Percentages in the ‘Goat isolates’ and ‘Kid isolates’ columns represent the proportion of each bacterial species isolated from goats or kids relative to the total number of isolates of that species

In the phenotypic test for production of ESBL, 28.26*%* (26/92) isolates were positive, with 15.38*%* (4/26) from kids’ rectal swab and 84.62*%* (22/26) from goats. Among the 22 positive isolates from goats, 54.54*%* (12/22) were *E coli*, 13.63*%* (3/22) *E. asburiae*, 9.09*%* (2/22) *E. cloacae*, 9.09% (2/22) *K. pneumoniae*, 9.09% (2/22) *Cronobacter* sp., and 4.54% (1/22) *K. variicola*. Of the 4 positive isolates from kids, 75% (3/4) were *E. coli* and 25% (1/4) was *E. cloacae*. Detection of genotypic production of ESBL and virulence factors are described in Table 2.

**Table 2:** Distribution of ESBL resistance and virulence genes among *Enterobacteriaceae* isolates from dairy goats and kids

Type	Genes	Goat isolates (71)			Kid isolates (21)
		E. coli (57)	K. variicola (2)	E.cloacae (2)	E. coli (19)
**ESBL**	*blaSHV*	-	1 (50%)	-	-
	*blaTEM*	1 (1.75%)	-	-	-
	*blaCTX-M*	-	-	-	-
**Virulence**	*hlyA* and *stx1*	6 (10.53%)	-	-	5 (26.32%)
	*hlyA*	6 (10.53%)	-	1 (50.00%)	2 (10.53%)
	*stx2*	4 (7.02%)	-	1 (50.00%)	2 (10.53%)
	*hlyA* and *stx2*	2 (3.51%)	-	-	-
	*stx1*	-	-	-	1 (5.26%)
	*eaeA*	-	-	-	-

Percentages represent the proportion of isolates positive for each gene relative to the total number of isolates of the same bacterial species and host group (n)

## Discussion

No *Enterobacteriaceae* were isolated from milk samples. Although this finding may suggest a low involvement of enteric Gram-negative bacteria in mastitis in the studied herds, it should be interpreted with caution, as our sampling focused on culture-based detection and included only a limited number of farms. Nevertheless, the difference between the fecal and milk microbiological profiles reinforces the notion that fecal carriage does not necessarily translate to intramammary infection.

*E. coli* was the most prevalent bacterium in the fecal samples, which agrees with Azevedo (2012), who reported the isolation of this species in 100% of samples from healthy sheep and goats in Petrolina, Pernambuco, Brazil. Hamzah et al. (2020) also described *E. coli* strains from ruminants as one of the main contaminants of drinking water in rural areas of Kenya.

*Enterobacter* and *Klebsiella* species have a high mutation capacity, favoring the acquisition and dissemination of genes responsible for antibiotic resistance (Rolbiecki et al. 2021; Annavajhala et al. 2019). In addition, *Klebsiella* spp. may cause diarrhea in livestock, representing a public health risk due to the spread of virulent strains (Mario et al. 2023).

*Cronobacter* sp., commensal microorganisms of the ruminant gastrointestinal tract, have also been reported in cereal samples in Brazil (Brandão et al. 2017), in artisanal cheeses made with raw milk in southern Brazil (Santos et al. 2023), and in goat milk powder factories in China (Fang et al. 2015), reinforcing their zoonotic potential and public health relevance.

The identification of ESBL-producing bacteria in healthy herds represents an important sanitary indicator. The use of antimicrobials in veterinary medicine contributes to the emergence and dissemination of resistant strains (Singh et al. 2024). Moreover, up to 90% of antimicrobials used in livestock production are released into the environment, intensifying the selection of resistant microorganisms (Karwowska 2024).

Although our study did not evaluate antimicrobial use on farms or environmental dissemination routes, the identification of ESBL-producing *E. coli*,* K. pneumoniae*,* Enterobacter* spp. and *Cronobacter* spp. indicates that healthy small ruminants may act as reservoirs of clinically relevant resistance determinants, as previously described for livestock in other settings. This is important because fecal shedding of resistant *Enterobacteriaceae* has been associated with environmental persistence and potential contamination of animal-derived products, as reported in other regions (Rolbiecki et al. 2021; Ewers et al. 2021). The World Health Organization (WHO 2024) classifies ESBL-producing *Enterobacteriaceae*, including *K. pneumoniae* and *Enterobacter* spp., as critical priority pathogens due to their role in the dissemination of antimicrobial resistance.

The detection of virulence genes, particularly *stx1*,* stx2* and *hlyA*, in *E. coli* isolates from both goats and kids further highlights the pathogenic potential of strains circulating in small ruminant herds. Although the presence of these genes does not necessarily indicate disease, their detection in healthy animals has been described elsewhere and suggests that asymptomatic carriers may serve as reservoirs for strains that can cause diarrhea or systemic disease under certain conditions (Yang et al. 2023; Persad et al. 2022; Mishra et al. 2020).

This study has some limitations, including its cross-sectional design, the focus on a single geographic region, and the use of culture-based methods. Additionally, antimicrobial usage history and genomic features of the isolates were not assessed. Even so, the findings provide important insights into the distribution of *Enterobacteriaceae*, ESBL phenotypes, and virulence genes in healthy goats in northeastern Brazil, reinforcing the relevance of continued surveillance within a One Health context.

In conclusion, the presence of ESBL-producing and virulent *Enterobacteriaceae* in fecal samples from healthy goats and kids suggests that these animals may act as reservoirs of resistance and virulence genes. Although no *Enterobacteriaceae* were isolated from milk, the fecal findings highlight the importance of monitoring small ruminant herds. Even without evaluating environmental or food-chain transmission, the results reinforce the need to include livestock in antimicrobial resistance surveillance. Future studies with genomic and environmental approaches will help clarify the epidemiology and potential public health impacts.

## Data Availability

No datasets were generated or analysed during the current study.
